# Prediction of Multimorbidity Network Evolution in Middle-Aged and Elderly Population Based on CE-GCN

**DOI:** 10.1007/s12539-024-00685-0

**Published:** 2025-02-10

**Authors:** Yushi Che, Yiqiao Wang

**Affiliations:** 1 School of Traditional Chinese Medicine, Beijing University of Chinese Medicine, Beijing, 100029 China; 2 School of Mathematics, Statistics and Mechanics, Beijing University of Technology, Beijing, 100124 China

**Keywords:** Multimorbidity, Link prediction, Evolving network

## Abstract

**Purpose:**

With the evolving disease spectrum, chronic diseases have emerged as a primary burden and a leading cause of mortality. Due to the aging population and the nature of chronic illnesses, patients often suffer from multimorbidity. Predicting the likelihood of these patients developing specific diseases in the future based on their current health status and age factors is a crucial task in multimorbidity research.

**Methods:**

We propose an algorithm, CE-GCN, which integrates age sequence and embeds Graph Convolutional Network (GCN) into Gated Recurrent Unit (GRU), utilizing the topological feature of network common neighbors to predict links in dynamic complex networks. First, we constructed a disease evolution network spanning from ages 45 to 90 years old using disease information from 3333 patients. Then, we introduced an innovative approach for link prediction aimed at uncovering relationships between various diseases. This method takes into account patients’ age to construct the evolutionary structure of the disease network, thereby predicting the connections between chronic diseases.

**Results:**

Results from experiments conducted on real networks indicate that our model surpasses others regarding both MRR and MAP. The proposed method accurately reveals associations between diseases and effectively captures future disease risks.

**Conclusion:**

Our model can serve as an objective and convenient computer-aided tool to identify hidden relationships between diseases in order to assist healthcare professionals in taking early disease interventions, which can substantially lower the costs associated with treating multimorbidity and enhance the quality of life for patients suffering from chronic conditions.

**Graphical Abstract:**

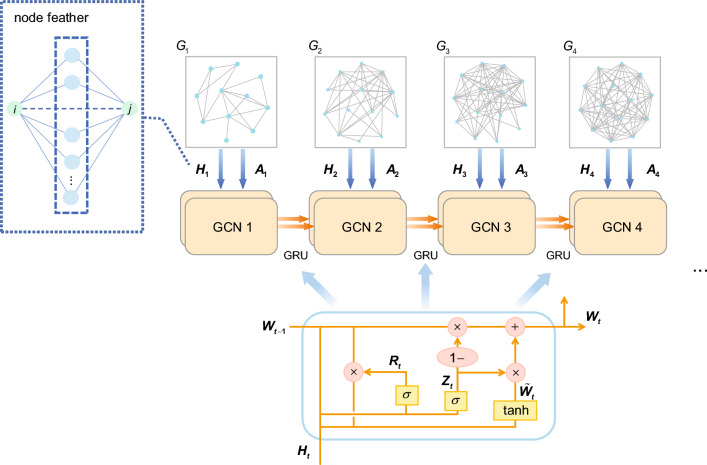

## Introduction

More than a third of the global disease burden is attributed to chronic diseases. The 2021 World Health Statistics from the World Health Organization (WHO) revealed that chronic diseases represented 7 out of the top 10 causes of death globally, compared to 4 in 2000. The number of premature deaths (deaths between the ages of 30 and 70) with chronic diseases was approximately 17 million in 2019 [1]. Chronic diseases are becoming the biggest challenge to global health. Chronic conditions are generally long-term and stem from a blend of genetic, physiological, environmental, and behavioral influences. With the aging population and the inherent nature of chronic illnesses, it is common for patients to suffer from several chronic ailments at once. That is the coexistence of two or more chronic diseases in the same individual, which is called multimorbidity [2].

Within the healthcare field, multimorbidity is a vital area of research. The prevalence of multimorbidity is increasing year by year. A large study highlighted a significantly increase in multimorbidity as age increases. Multimorbidity is present in most people aged 65 and older, with nearly 23$\%$ of the population suffering from more than one chronic disease [3]. Studies in China have revealed that around 65.5$\%$ of the middle-aged and senior individuals suffer from multimorbidity, which has emerged as a serious public health challenge. With changes in the disease spectrum, chronic diseases have become a major burden of disease and a leading cause of death. Despite the marked increase in life expectancy, there is a rapid rise in the number of people with or at risk for chronic diseases [4].

Indeed, the increasing prevalence of multimorbidity is a major challenge for patients, medical staff, and healthcare institutions. On the one hand, for patients, the cost and complications of treating multimorbidity are increasing exponentially. Patients with multimorbidity may also have limited mobility. To control these conditions, a considerable amount of medication is often needed, but this can lead to side effects that adversely affect quality of life. The strain of living with such conditions also often leads to depression. On the other hand, for medical professionals and institutions, the treatment and care of multimorbidity is highly complex. The National Health Service (NHS) in England established the Multimorbidity Expert Reference Group (MMERG) to develop an evidence base for preventing and managing multiple long-term conditions. This group, composed of clinicians, academics, statisticians, data leaders, and economists, aims to improve care outcomes and experiences. They assert that an integrated care system is crucial for achieving effective multimorbidity management. The system’s infrastructure integrates community, primary, and secondary care services with local government and the voluntary sector, enabling more efficient care coordination for multiple conditions. Guided by integrated datasets and population health management tools, innovative personalized care pathways are being developed across providers using population segmentation and risk stratification. The Australian Institute of Health and Welfare has introduced measures to enhance care coordination for patients with multimorbidity. These include providing multidisciplinary care for chronic disease management through general practitioners, developing medical guidelines (such as those for managing coexisting asthma and chronic obstructive pulmonary disease), and reviewing medication appropriateness for patients. Additionally, the MyMedicare website offers a voluntary registration model that allows patients to receive more continuous care. They are also trialing Health Care Homes, which enable patients to sign up with a specific general practice or Aboriginal Community Controlled Health Service for integrated care and team-based support. However, both models require substantial investment in infrastructure and training, which may pose challenges for countries with less healthcare funding. Based on the characteristics of the two countries mentioned above, China has proposed a strategy suited to its national circumstances. The 2023 China Expert Consensus on Elderly Multimorbidity Management recommends a strategy tailored to China’s national conditions and the unique characteristics of its elderly population. Health status stratification is used to set different management goals for elderly multimorbidity patients. Comprehensive geriatric assessments evaluate the impact of multimorbidity and identify patients’ specific needs, guiding intervention plans. Due to the time-intensive nature of these assessments, combining them with targeted screenings can enhance clinical efficiency. Screening helps quickly identify at-risk elderly individuals. Furthermore, due to traditional medical research focusing on the treatment of a single disease, there is uncertainty about the ability to effectively treat patients with multimorbidity [5]. Additionally, those suffering from multimorbidity could have a higher level of overall vulnerability to diseases and decreased resistance to acute health challenges. The interplay of these diseases leads to a complex pattern of health care utilization. At the same time, multimorbidity leads to an increased likelihood of referral, which also means increased health care costs and more fragmented care for the disease. Patients’ health is harmed by the poor integration, communication and coordination of disease care [6].

WHO argues that multimorbidity has become more common than uncommon among individuals with chronic conditions. Regarding their health care issues, preventive care is a key area to explore [2]. Today, the diagnoses made by medical practitioners for chronic diseases are generally based on a number of specific medical parameters. Therefore, most interventions tend to occur after the onset of the chronic disease and rarely begin at the early signs of the disease [7]. Moreover, for the treatment of multiple chronic diseases, accurately predicting disease progression can lead to better treatment decisions by physicians. In addition, the main purpose of forecasting disease is to be able to prevent it, because it is easier to prevent a chronic disease than to reverse it [8]. Therefore, the identification of early multimorbidity and risks in healthcare should be explored more proactively rather than reacting after the diseases have occurred [9, 10].

To address the prevention of multimorbidity, predicting the evolutionary trends of multimorbidity is significant. It can be considered as an evolution of a complex system of multimorbidity. Complex systems are often modeled as complex networks before analysis. In such models, nodes represent the individuals, and edges signify the relationships or interactions among these individuals. Currently, the main methods for analyzing complex networks include DeepWalk [11] based on network topology information, HOPE [12] based on matrix decomposition, and GCN [13] based on neural networks, among others. In particular, networks utilizing topology measurements are commonly employed due to their generalized nature and the fact that they do not depend on specific details related to the node contents.

However, most of the above methods are applied to static networks. In practical applications, networks generally evolve over time. For example, consider the occurrence of new diseases or new multimorbidities in a disease network. Thus, real-world networks generally have temporal characteristics. Static network analysis does not reflect the temporal characteristics in dynamic networks, nor does it accommodate the evolving nature of nodes and their relationships. This deficiency leads to learning outcomes that lack authenticity and dynamism, often resulting in less effective applications. Nevertheless, a key similarity exists, as both dynamic and static graphs must be transformed into graph data for analysis. Therefore, the analysis of many dynamic networks can be inspired by the analysis of static networks.

As time progresses, the network changes continuously because nodes or edges in the network may be added or removed [14]. Predicting the links between nodes in the network is challenging due to weak correlations, uncertainties, and the non-smooth characteristics of time and space. The dynamic network is commonly represented as a series of successive network snapshots at first. Then, a predictive model is used to learn fixed-size embeddings for each node in these snapshots, and link predictions are mapped to the embedding space [15]. Real-world networks often demonstrate complex evolutionary dynamics, which adds to the difficulty of predicting potential linkages over time. Many studies have explored the properties, evolutionary processes, and functions of networks. [16]. In some ways, adopting an appropriate link prediction approach to estimate the likelihood of links between two nodes in a real-world dynamic network provides substantial theoretical and practical benefits.

In dynamic networks, link prediction aims to predict the changes in network connections over time. Recently, several studies have predicted links in networks using time intervals. Nguyen et al. [17] established a framework that integrates temporal data into random walk-based embedding approaches to learn network representations that reflect the temporal characteristics of dynamic networks. Kaya and Poyraz [7] designed the disease network’s evolving structure according to the ages of the patients. The similarity score between nodes is determined based on the evolution of the dynamic network, which is then used to estimate the edge probability between candidate node pairs. Based on the concept of temporal events, Soares and Prudencio [18] proposed a new link prediction method. The representative score for a pair of nodes is updated according to the establishment, maintenance, or disruption of relationships between them during consecutive time periods. Many network prediction models employ GCN developed by Kipf and Welling [13]. GCN can directly process graphs and capitalize on their structural information. Dong et al. [19] established a disease relationship network based on two databases. They converted the multimorbidity prediction task into a link prediction task. In the prediction process, they employed GCN model to incorporate chosen phenotypes into the disease network for predicting multimorbidity. GCN is mainly designed to capture features in static networks, whereas Recurrent Neural Network (RNN) is adept at capturing features in time-series data. Given these characteristics, combining the two models enables effective feature extraction in dynamic graphs. For example, GCRN model, designed by Seo et al. [20], addresses modeling and prediction for time-varying graph-based data. By combining GCN and Long Short-Term Memory network (LSTM), this model can identify spatial structures and dynamic patterns simultaneously. Chang et al. [21] proposed a GCN-LSTM model incorporating an attention mechanism. They first extracted features from raw EEG signals to construct graph data, then used GCN-LSTM model to capture spatiotemporal features while assigning weights to spatial information through the attention mechanism. The model significantly improved emotion recognition performance on SEED dataset. Similarly, Reid et al. [22] proposed a model combining GCN and LSTM to predict the spatiotemporal incidence of COVID-19 in the Metropolitan Region of Chile. In this model, GCN addresses spatial correlations between neighboring cities, and LSTM is employed for time-varying predictions. However, GCRN trains one single Graph Neural Network (GNN) model which is learned for all graphs on the temporal axis. The model ignores the internal connection between the structural features of the graph and the temporal features. EvolveGCN [23] has improved the above problems. To capture the graph sequence’s dynamics, the approach uses an RNN to evolve GCN parameters. They suggested using RNN regulates GCN model at each time step. The model effectively performed adaptation. Liang et al. [24] employed EvolveGCN model to capture the dynamics of brain network sequences for predicting the progression of Alzheimer’s disease. Additionally, Graphormer [25] is a model specifically designed for graph data, leveraging Transformer architecture to improve graph representation learning. Given the success of Transformer in handling sequential data, particularly in research fields such as computer vision have aimed to extend this approach to graph data. To achieve this, Graphormer incorporates graph structural information, such as distance metrics between nodes, into Transformer’s self-attention mechanism to better capture the structural features of graphs. Hu et al. [26] proposed BrainNPT neural network model, which is based on Transformer and uses GNN to embed brain region networks as vectors. Transformer is then used to capture effective representations of these brain networks. This approach ranks the connectivity strength of brain regions and identifies abnormal connectivity areas in patients with autism. This research further demonstrates the effectiveness of applying Transformer to dynamic graph data.

This research explored how multimorbidity develops in middle-aged and older adults as they age. We have designed a new model, CE-GCN, which can predict the risk of multimorbidity development. We expect to be able to take timely preventive treatment in the early stages of the disease. Firstly, constructing the multimorbidity network is based on whether two different chronic diseases occur simultaneously. In this network, nodes indicate diseases and edges indicate multimorbidity that one patient suffers from. Secondly, age is added to the model as a temporal feature and the disease network changes with age. We assumed that the higher the count of common neighbors between two diseases, the greater their likelihood of being connected in the future. In addition, the past co-morbidity of the two diseases was also considered. They impact on the future probability of co-morbidity between the two diseases. Therefore, predicting multimorbidity can be seen as an application of link prediction. Finally, we considered a GCN algorithm based on age sequences and used network topology features for link prediction in dynamic complex networks. To reduce parameters and improve training efficiency, we proposed a recurrent model using Gated Recurrent Unit (GRU) and embedded GCN into GRU’s input gate. This integration allows GRU to learn dynamics from extracted node features in evolving network parameters, enabling GCN to capture the structural characteristics of the dynamic network while sharing parameters with GRU. The new model we propose enables the identification of hidden relationships between diseases, rather than simply extracting the links between existing diseases.

## Materials and Method

### Network Definition

**Fig. 1:**
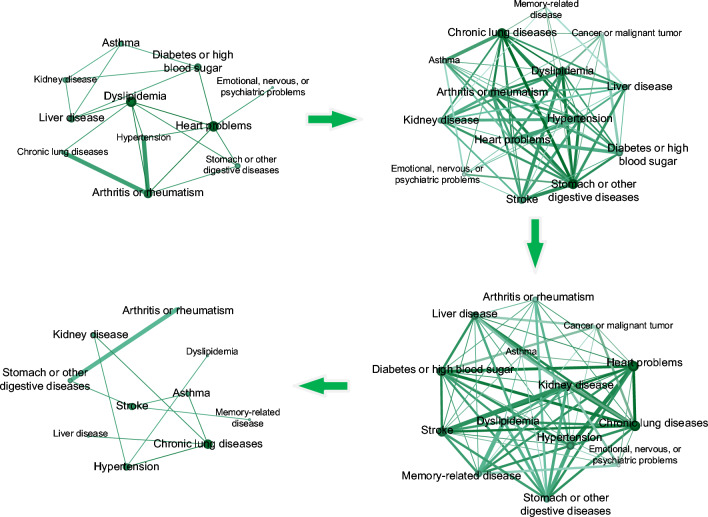
The figure of chronic diseases evolution. The four networks are presented at the age stages of 45, 60, 75, and 90. The set of graphs also roughly shows the multimorbidity condition of middle-aged, young-aged, old, and oldest-old. In order to show the number of patients, graphs are weighted with edges, where the thicker the edge, the higher the number of patients with both diseases

In this study, we constructed a disease evolution network using the China Health and Retirement Longitudinal Study (CHARLS) database, which includes 16 common chronic diseases. The evolution of multimorbidity network is defined as $G=\left\{ G_1, G_2, \ldots , G_t, \ldots \right\}$, where each $G_t=\left( V_t, E_t\right)$ represents the state of graph at time *t*. We use subscript *t* to denote the age index. $V_t$ and $E_t$ are the set of vertices and edges respectively. $V_t=\left\{ v_{1 t}, v_{2 t}, \ldots , v_{n t}\right\}$ represents different chronic diseases categories under time *t*. $E_t$ denotes the simultaneous occurrence of two diseases within a patient at time *t*. Specifically, if diseases $v_{i t}$ and $v_{j t}$ co-occur at time *t*, we set the corresponding element $e_{i j t}$ in $E_t$ to 1. If two diseases do not co-occur at time *t*, the value of $e_{i j t}$ is set to 0. The rules of edges are as follows:

$$\begin{aligned} {\left\{ \begin{array}{l}e_{i j t}=1, \text{ a } \text{ patient } \text{ suffered } \text{ from } \text{ two } \text{ chronic } \text{ diseases } \\ e_{i j t}=0, \text{ otherwise }\end{array}\right. } \end{aligned}$$

where $e_{i j t} \in E_t$. The disease network generated by this approach is shown in Fig. 1.

We captured four age groups for a rough demonstration. In particular, the age stage is selected according to the United Nations World Health Organization’s age classification criteria for different population stages: people aged 45–59 as “middle-aged”, 60–74 as “young-old”, 75–89 as “old”, and those 90 and above as the “oldest-old”. Sequential snapshots are created by age sequences. We divided the disease evolution network into snapshots, each capturing the disease status of patients at various ages.

### Network Architecture

**Fig. 2:**
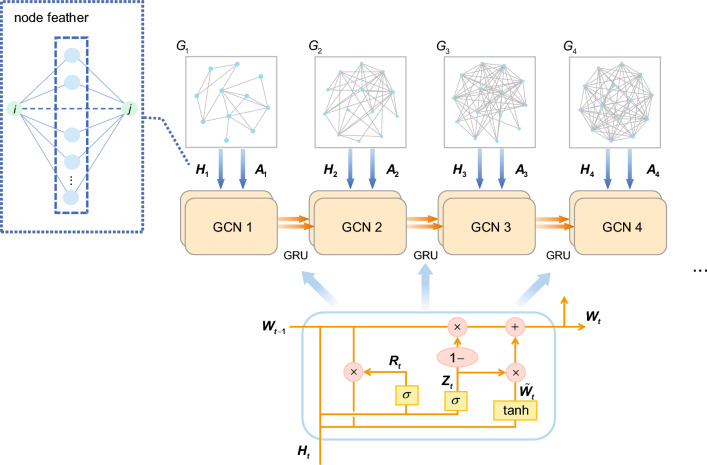
The construction of CE-GCN

Based on the above chronic disease evolution network, we propose a model for predicting evolution networks using a dynamic GCN with network topology metrics. The model framework is shown in Fig. 2.

To capture the dynamic graph structure, GRU is used as the recurrent model to update GCN parameters, enabling GCN to more effectively capture the features at each time step of the dynamic graph. In this evolution, we focus on the structural characteristics of the nodes and their neighboring nodes. Capturing temporal information enables us to predict node changes and derive their future characteristics.

#### GCN Based on Common Neighbors

We apply a GCN to capture the structural features. In $G_t$, the adjacency matrix $\varvec{A_t}$ and the node embedding matrix $\varvec{H_{t (l)}}$ are considered as inputs, and the latter is updated to $\varvec{H_{t (l+1)}}$ as an output using the weight matrix $\varvec{W_{t (l)}}$. The subscript (*l*) denotes the GCN layer index. The rules of propagation between the layers of the graph convolution as follows:

1 $$\begin{aligned} {\varvec{H_{t (l+1)}}=\sigma \left( \varvec{\tilde{D_t}}^{-\frac{1}{2}} \varvec{\tilde{A_t}} \varvec{\tilde{D_t}}^{-\frac{1}{2}} \varvec{H_{t (l)}} \varvec{W_{t (l)}}\right) } \end{aligned}$$

$\varvec{\tilde{A_t}}$ represents is the adjacency matrix with added self-loops. $\varvec{\tilde{D_t}}$ is the degree matrix of $\varvec{\tilde{A_t}}$. $\sigma (\cdot )$ means the activation function.

The initial embedding matrix is derived from the node features, represented as ${\varvec{H_{t (0)}}}=\varvec{F_t}$ where $\varvec{F_t} \in \mathbb {R}^{n \times d}$, with each row of $\varvec{F_t}$ corresponding to a *d*-dimensional feature vector for each node. In the process of constructing node features, we performed a dot product on the adjacency matrix of the network, resulting in $\varvec{A_t}^2$. In the dot product matrix, $a_{i i}$ represents the degree of node *i*, and $a_{i j}$ represents the number of common neighbors between nodes *i* and *j*. We combined these two features. While using only the node degree method can effectively capture the potential relationships within the entire network, it lacks local information. Therefore, we included the number of common neighbors in the node features. Calculating node similarity based on common neighbors to predict future connections is a widely used method [27–29]. This method captures local topological information, addressing the aforementioned shortcomings. Next, we constructed a onehot encoding. When using one-hot encoding, the length of each vector equals the total number of node features, with only one corresponding position set to 1, while all others are set to 0. The reason for constructing node features with one-hot encoding is that it provides discrete network information. This approach prevents the introduction of ordinal relationships during encoding and avoids overlook certain nodes due to numerical feature scaling. Each distinct degree corresponds to a dimension in the feature vector, allowing the model to handle this information separately. As a result, the model can better adapt and recognize the impact of this information on prediction results.

2 $$\begin{aligned} {f_t(i)=\varvec{A_t}^2 \cdot \varvec{M}^{n \times 1}} \end{aligned}$$

where $\varvec{M}$ is a column vector of size *n* with all elements equal to 1.

#### Node Evolution

We use GRU to dynamically adjust GCN parameters, allowing GCN to adaptively capture structural features in evolving graphs. With its suitability for capturing temporal information, GRU learns node feature behaviors across snapshots, extracts stable connection features, and anticipates node connections in future snapshots. The core of the method is to update $\varvec{W_{t (l)}}$ based on current and historical node information. GRU consists of an update gate and a reset gate. The update gate reflects the influence of previous state information. A higher value for the update gate brings more information from the past into the present state. The reset gate, on the other hand, controls how much previous state information is written into the current candidate set $\varvec{\tilde{W_t}}$. A lower reset gate value means less information from the previous state is retained, signifying a greater degree of forgetting past information. In GRU, we use the weights from the previous time slice with the current node features to train the weights used in GCN. The calculation method is as follows:

3 $$\begin{aligned} & \varvec{W_{t (l)}}={\text {GRU}}\left( \varvec{H_{t (l)}}, \varvec{W_{{t-1}{(l)}}}\right) \end{aligned}$$

4 $$\begin{aligned} & \varvec{Z_t}={\text {sigmoid}}\left( {\varvec{U_{Z}} \varvec{H_{t (l)}}+\varvec{C_{Z}} \varvec{W_{{t-1} {(l)}}}+\varvec{B_{Z}}}\right) \end{aligned}$$

5 $$\begin{aligned} & \varvec{R_t}={\text {sigmoid}}\left( {\varvec{U_{R}} \varvec{H_{t (l)}}+\varvec{C_{R}} \varvec{W_{{t-1} {(l)}}}+\varvec{B_{R}}}\right) \end{aligned}$$

6 $$\begin{aligned} & \varvec{\tilde{W}_t}=\tanh \left( {\varvec{U_{W}} \varvec{H_{t (l)}}+\varvec{C_{W}}\left( \varvec{R_t} \circ \varvec{W_{{t-1} {(l)}}}\right) +\varvec{B_{W}}}\right) \end{aligned}$$

7 $$\begin{aligned} & \varvec{W_{t (l)}}=\left( 1-\varvec{Z_t}\right) \circ \varvec{W_{{t-1} {(l)}}}+\varvec{Z_t} \circ \varvec{\tilde{W}_t} \end{aligned}$$

$\varvec{U_{Z}}$, $\varvec{C_{Z}}$, $\varvec{U_{R}}$, $\varvec{C_{R}}$, $\varvec{U_{W}}$ and $\varvec{C_{W}}$ are the weight matrices. $\varvec{B_{Z}}$, $\varvec{B_{R}}$ and $\varvec{B_{W}}$ are the bias vector. $\circ$ denotes Hadamard product.

#### The Task of Link Prediction

The embeddings of nodes *i* and *j* prior to time $t+1$ are used to predict whether an edge will exist at time $t+1$. We aggregate the feature values learned by node *i* and node *j* and then use Multilayer Perceptron (MLP) to output the probability of node connections. The process of constructing the loss function uses the optimized cross-entropy method of Pareja et al. [23].

## Experiment

### Dataset

Since the core of this study is to predict the evolution of multiple chronic diseases in a middle-aged and elderly population, we focused on analyzing the relationships among common chronic diseases in this group. For this study, we analyzed the data from the 2018 China Health and Retirement Longitudinal Study (CHARLS 2018). The database included data on age and chronic diseases in middle-aged and older adults. The project set up a public database for Chinese individuals aged 45 and older. The CHARLS survey included 150 counties/districts and 450 rural and urban communities across China, surveying 17,708 individuals [30]. The data used in this study consists of chronic disease records for individuals aged 45–90, as tracked in CHARLS 2018. To construct the evolving disease network, we subjected this dataset to a data cleaning process. This involved excluding individuals without chronic diseases or with only one chronic disease. Following these processes, the study ultimately collected data on 3333 patients and 14 diseases. The prevalence of 14 chronic diseases is shown in Table 1.

**Table 1:** The prevalence of 14 chronic diseases among the middle-aged and elderly patients

Disease categories	Number of patients
Dyslipidemia	1235 (37.05%)
Hypertension	1193 (35.79%)
Stomach or other digestive diseases (except for tumor or cancer)	892 (26.76%)
Heart attack, coronary heart disease, angina, or other heart problems	864 (25.92%)
Arthritis or rheumatism	799 (23.97%)
Diabetes or high blood sugar	681 (20.43%)
Chronic lung diseases ( excluding tumors, or cancer)	639 (19.17%)
Stroke	611 (18.33%)
Kidney disease (except for tumor or cancer)	489 (14.67%)
Liver disease (except fatty liver, tumors, and cancer)	417 (12.51%)
Memory-related disease	325 (9.75%)
Asthma	317 (9.51%)
Emotional, nervous, or psychiatric problems	168 (5.04%)
Cancer or malignant tumor (excluding minor skin cancers)	147 (4.41%)

### Baseline

To verify the validity of CE-GCN, we will compare it with the following four baselines.

*GCN* [13]. Without temporal modeling, a single GCN model is used for all time steps, with the loss accumulated over the time axis. GCN is a network structure feature extractor in CE-GCN and was tested as a modular unit.

*GCN-LSTM* [20]. This model combines GCN with LSTM model, specifically designed to handle graph data with temporal dynamics. It uses GCN to extract structural features of nodes for node embeddings and then inputs these features into an LSTM to capture the dynamic relationships of node features.

*EvolveGCN* [23]. This method generates node embeddings by combining a fixed GCN for capturing node features and a GRU for evolving temporal relationships. GCN extracts node information, and GRU evolves the parameters to capture time-series patterns.

*Graphormer* [25]. It is a graph data processing model based on Transformer architecture, designed for efficient graph representation learning. It enhances the ability to capture structural features of graphs by integrating graph structure information into Transformer’s self-attention mechanism.

### Evaluation Tasks and Metrics

#### Mean Average Precision (MAP)

MAP is a crucial metric for evaluating the effectiveness of information retrieval systems or ranking models. It measures the overall performance of a system across multiple queries, particularly assessing the relevance of multiple relevant results for each query. MAP integrates accuracy and recall to offer a global performance index for the algorithm. MAP evaluates both the relevance of recommended items and the system’s ability to rank more relevant items higher in the results. Higher MAP values indicate superior retrieval performance across multiple queries. AP is an approximation of the area under the precision-recall (PR) curve, a value between 0 and 1. PR curve is a curve plotted with precision (*P*) as the vertical coordinate and recall (*R*) as the horizontal coordinate. Mathematically, we formulate them as the following:

8 $$\begin{aligned} {\textrm{AP}=\int _0^1 { P(R)dR } } \end{aligned}$$

9 $$\begin{aligned} {\textrm{MAP}=\frac{1}{K} \sum _{k=1}^K \textrm{AP}_k} \end{aligned}$$

where MAP is the average value of AP, and *K* denotes the number of categories of AP.

#### Mean Reciprocal Rank (MRR)

MRR is another measure for assessing the performance of information retrieval systems and ranking models. It measures the position of the first relevant result in the returned ranking. Higher MRR values indicate better system or model performance in ranking relevant results. This metric focuses on whether the target term is at the top of the model calculation list.

10 $$\begin{aligned} {\textrm{MRR}=\frac{1}{S} \sum _{i=1}^S \frac{1}{q_i}} \end{aligned}$$

where *S* denotes the number of samples, $q_i$ denotes the position where item *i* appears in the list, and if it does not appear then $\frac{1}{q_i}=0$.

## Results

In this study, five models are designed and implemented, and their performance was tested through link prediction experiments on the multimorbidity network. These experiments were designed to compare CE-GCN’s performance with that of existing models for predicting future multimorbidity occurrences. During the experimental process, CE-GCN’s computational complexity was evaluated on CHARLS 2018 dataset. The total number of trainable parameters in CE-GCN is 160,076, with an estimated storage requirement of 0.61 MB. The average training and testing time is approximately 1.53 s and 0.36 s, respectively. To estimate the computational complexity on other large datasets, we derived the time complexity based on the structure of CE-GCN. The time complexity of the graph convolution module depends on the number of nodes *n* in the graph, the number of edges *m*, the dimension of the input features *d*, and the hidden layer dimension *h* in each layer. We used a standard GCN layer, so the computational complexity is $\mathcal {O}(m \cdot d+n \cdot d \cdot h)$. CE-GCN uses GRU to update GCN weights over time. Assuming the model trains on a dynamic graph over *T* time steps, the temporal evolution complexity can be expressed as $\mathcal {O}(T \cdot h^2)$. The combined complexity of the two modules is $\mathcal {O}(T \cdot (m \cdot d+n \cdot d \cdot h+h^2))$. Table 2 presents the prediction results of five models under various experimental conditions.  The bold values represent the optimal results in the performance evaluation. The experimental results indicate that CE-GCN surpasses other models in both MAP and MRR, achieving improvements of 0.0083 and 0.0621, respectively, over the second-best algorithm. The superior performance of CE-GCN in the test results suggests that it excels in both overall performance and the accuracy of top-ranked predictions. Specifically, the increase in MAP indicates that the new model ranks relevant links higher, demonstrating better ranking performance across multiple prediction tasks. The improvement in MRR suggests that the model identifies correct multimorbidity associations more quickly and at earlier stages. These findings imply that existing models exhibit relatively low accuracy in predicting the formation of new relationships in the multimorbidity network, whereas our model identifies potential disease associations more effectively, demonstrating stronger predictive capability.

**Table 2:** The performance of different models

Model	MAP	MRR
GCN	0.3891	0.2980
GCN-LSTM	0.4304	0.2000
EvolveGCN	0.3619	0.3112
Graphormer	0.4257	0.2500
CE-GCN	**0**.**4387**	**0**.**3733**

**Fig. 3:**
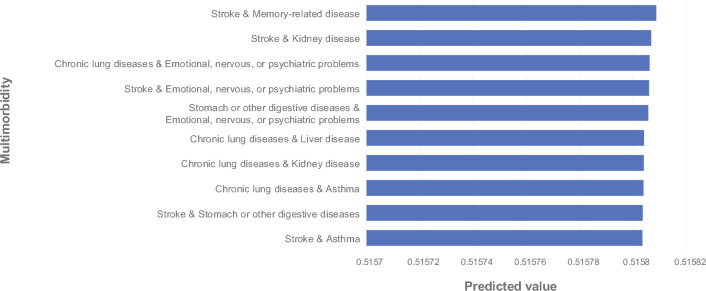
The top 10 combinations of chronic diseases with the highest prediction scores

The data for this study comes from real research data on the older adults in China. We combined network structure with temporal factors to identify potential future occurrences of multimorbidity. Our model is trained on the multimorbidity network structure features of younger age groups, using age as a time series to predict new multimorbidity relationships at the next age stage. In previous studies, some researchers have proposed relying on the overall disease network structure to infer the progression of multimorbidity [31]. Our model also considers the impact of the local structure of the network on prediction results. The more multimorbidity nodes two diseases share, the more likely they are to form an edge in this disease network, indicating that they may be influenced by the same risk factors. Research has shown that if two diseases share many of the same multimorbidity, this suggests that they may have significant overlap in etiology and disease mechanisms, which could explain this phenomenon [32], for example, metabolic disorders or immune system dysfunctions. [33] Additionally, our model also integrates age as a time series factor, and as age increases, multimorbidity networks evolve accordingly. Figure 3 displays the predictions of multimorbidity occurrences made by our model, where we provide both the actual associations of diseases in later stages and the predicted results. A higher prediction value for a disease pair indicates a stronger hidden relationship in earlier stages, suggesting a higher likelihood of future co-occurrence. We selected some combinations of chronic diseases to validate the predictive ability of the model, showing the top 10 chronic disease combinations with the highest prediction scores.

Among the five groups of multimorbidity with high prediction scores, stroke and memory-related disease can be considered to correlate strongly. In the disease evolution graph, we can see multimorbidity networks at different ages. At earlier age stages, there is no direct edge between stroke and memory-related disease, although they share many common neighbors. Most diseases related to memory form multimorbidity relationships with stroke. As age progresses, a direct edge appears between stroke and memory-related disease. Cognitive impairment and memory dysfunction after stroke were recognized in the stroke study as common symptoms that significantly affect the quality of life of survivors. Nearly one in three stroke patients develop dementia within the first year after their stroke [34]. This is because stroke is primarily an abnormality of the blood vessels in the brain, such as stroke within the middle or anterior cerebral artery territory, which can cause memory loss. Studies have shown that, depending on the location of brain damage and the severity of stroke, there may be one or more types of memory functionality loss. Ultimately, patients end up with memory decline and loss [35]. In addition, Alzheimer’s disease, a disease with severe memory loss, has a certain probability of worsening as the frequency of stroke increases.

The group of multimorbidity, stroke and kidney disease, was shown not to be in the actual link. However, from the multimorbidity network, we can see that the edge between stroke and kidney disease gradually thickens in the 45–75 age group, indicating an increasing number of patients over the years. Chronic kidney disease (CKD) has been shown to raise the risk of both ischemic and hemorrhagic stroke. Stroke risk increases incrementally with CKD stages in a dose-response effect that is independent of other vascular diseases. The risk factors for stroke are quite similar to those for CKD, such as advancing age, elevated blood pressure, and increased blood glucose. In addition to the above risk factors that contribute to the co-occurrence of stroke and CKD, cerebrovascular disease is also related to CKD mechanisms, including platelet and coagulation dysfunction, increased risk of atrial fibrillation, and more [36].

Furthermore, three of the top five conditions listed in Fig. 3 were associated with psychiatric disorders. From the multimorbidity network, we can also observe that the appearance of nodes related to psychiatric disorders makes the multimorbidity network denser. A study indicates that patients with both physical and mental illnesses have a higher risk of active safety incidents [37]. These incidents include the side effects of polypharmacy, complications, and adverse outcomes related to healthcare services. Individuals with only physical multimorbidity have a lower risk of these incidents. It is critical to understand the mechanisms behind active safety incidents in order to guide the design of interventions for improving the safety of patients with multimorbidity. Mental health cannot be neglected. Patients with chronic diseases frequently experience negative emotions, including depression, anxiety, frustration, and anger [38]. Functional emotional responses are adaptive in nature. However, overreactive emotions may lead to dysfunction and exacerbate chronic disease. Patients with depression or anxiety multimorbidity exhibit a higher prevalence of co-morbidities and worse health status than those with chronic diseases only [39]. There may be more people with chronic illnesses who need help with emotional issues than we think. Huang et al. [40] found in their study that up to 53.6% of chronic patients in high emotional disturbance group. This highlights the need for more support to help patients adapt to life with chronic illness. In this study, it was shown that particular attention should be paid to patients with chronic lung diseases, stroke and stomach or other digestive diseases.

In addition, Fig. 3 shows that the co-morbidities associated with chronic lung diseases should be taken into account. From the multimorbidity network, it is evident that the chronic obstructive pulmonary disease (COPD) gradually becomes a central node with increasing age, indicating that the multimorbidity rate of this disease is rising over time. COPD has a significant impact on multimorbidity. The rising prevalence of multimorbidity over time can largely be attributed to the progression of COPD, with almost all COPD patients presenting with at least one other chronic disease [41, 42]. There is a need to focus on the progression of COPD with liver disease, kidney disease and asthma.

## Conclusions

Globally, we have to take into account the prevention and treatment of multimorbidity because the quality of life of these patients is threatened. The prediction of multimorbidity using a model in primary care could free up more time for community physicians or family physicians to prevent the occurrence of multiple chronic diseases. Therefore, we propose a multimorbidity evolution network and an effective link prediction method of disease evolution network. In CE-GCN, the age of the patient is used as a temporal attribute for the evolution of the multimorbidity network. We use GCN and GRU for learning the evolution of dynamic networks. Furthermore, the relationship between the occurrence of co-morbidities of the two diseases and their respective other co-morbidities is considered in the evolution of the multimorbidity. The result in our work illustrates that the performance of CE-GCN on the task of link prediction is better than that of other models.

The prediction results of CE-GCN will be a useful tool to improve the preventive and curative services. In clinical practice, healthcare professionals can use the multimorbidity prediction results provided by the model to identify patients who are more likely to develop specific multimorbidities earlier. They are able to start dual management of these diseases earlier to prevent multimorbidities at an early stage. For public health policymaking, predicting the multimorbidity trends enables public health authorities to implement interventions in advance. The government can conduct early screening for high-risk populations and design more targeted health education content. Additionally, understanding that certain multimorbidities are more likely to occur can help the government allocate healthcare resources more efficiently, such as screening equipment, specialists, and medical facilities. By predicting multimorbidities, it is possible to identify priorities for prevention and treatment in different healthcare settings and detect potential health issues. Early intervention not only makes it possible to reduce the overall burden of disease, but also help to promote the health of society as a whole. For example, according to the prediction results of CE-GCN, Chinese region should be more concerned about the development of stroke and emotional-psychological disorders in the elderly in this study. The government can focus on high-risk groups by providing more personalized prevention, medical treatment, and care in the region. Furthermore, people with co-occurring mental and physical health conditions are at significant life safety risk. Consequently, the Chinese government needs to consider incorporating mental health programs within the scope of primary healthcare.

The aim of this study is to predict the occurrence of multimorbidity in the middle-aged and elderly population in China. Considering that living environments in other countries differ from those in China, this may lead to differences in the prevalence of multimorbidity. In future research, we will consider including multimorbidity data from other regions and countries to further generalize multimorbidity prediction studies. In future research on multimorbidity prediction, it is recommended to continue using a graph model that considers each disease as a network node, where edges between nodes represent multimorbidity relationships between diseases. Based on this network structure, CE-GCN proposed in this study can be used for disease evolution analysis, predicting the likelihood and timing of multimorbidity occurring in the same patient, thereby forecasting complex disease interrelations. Additionally, future research can apply our model to explore the patterns of multimorbidity evolution in different populations. By constructing and analyzing multimorbidity networks according to various demographic characteristics, it is possible to predict potential trends in multimorbidity development across different populations, enriching the understanding of multimorbidity phenomena.

In this paper, we proposed a homogeneous network learning model. However, many networks of practical relevance are heterogeneous networks, such as those involving disease and patient characteristic networks. In the future, we plan to integrate other data types, such as physiological data and multi-omics data, to construct more complex heterogeneous networks. This will enrich the information sources of the model, enabling more comprehensive representation and analysis. Moreover, considering CE-GCN’s advantages in capturing time-series features, we will explore its application in real-time health monitoring, combining wearable devices and electronic health records (EHR) to achieve dynamic tracking and prediction of patients’ health conditions.

## Data Availability

The data that support the fndings of this study are available from the corresponding authors upon reasonable request.

## Code Availability

Code is available from the corresponding authors upon reasonable request.
